# Effect of leadership style and organizational climate on employees' food safety and hygiene behaviors in the institutional food service of schools

**DOI:** 10.1002/fsn3.1056

**Published:** 2019-05-07

**Authors:** Wen‐Hwa Ko, Hsiu‐yu Kang

**Affiliations:** ^1^ Department of Restaurant, Hotel and Institutional Management Fu‐Jen University New Taipei City Taiwan

**Keywords:** employee, food safety and hygiene behaviors, leadership style, organizational climate, school lunch

## Abstract

This study took the employees working in the institutional food service of schools in northern Taiwan as subjects to explore the influences of leadership style and organizational climate on food safety and hygiene behaviors. In this study, 400 questionnaires were distributed and 324 valid questionnaires were collected, for a valid return rate of 81%. The results showed that leadership style and organizational climate positively correlate with employees' behaviors with respect to food safety and hygiene. Transactional and transformational leadership have a significantly positive effect on organizational climate. Organizational climate has a significantly positive impact on employees' food safety and hygiene behaviors. Transactional and transformational leadership have a significantly positive effect on employees' food safety and hygiene behaviors, but the difference between the two factors is not obvious. Organizational climate has a complete mediating effect on the process of transactional and transformational leadership affecting food safety and hygiene behaviors.

## 1 INTRODUCTION

In any modern and highly competitive business environment, leadership is regarded as one of the most critical factors for the success of the company's future development (Mohan, 2000). At the same time, the leader is also an important factor affecting the success or failure of the organization (Bennis & Nanus, 1985). Bass (1985) points out that leaders influence 45%–65% of the success or failure of an organization. Andersen (2016) believes that leaders can motivate, inspire, and identify with their employees to facilitate employees completing their work and achieving the desired goals. Moreover, leaders use a variety of different leadership styles to inspire and motivate employees.

Transactional and transformational leaders, as proposed by Burns (1978), have received greater attention in recent years. There is a process of exchange of benefits between transactional leaders and their subordinates to realize their respective goals (Bass, 1990). Unlike transactional leaders, transformational leaders motivate employees to do things beyond their abilities and to refocus from personal interests in favor of group interests (Bass, 1990). Food safety deals with relevant inappropriate behaviors and conditions, such as inappropriate temperatures, poor hygiene habits, and cross‐contamination, which have a significant relationship with the occurrence of food‐borne illnesses. Such inappropriate behaviors and conditions are caused by employees failing to comply with food safety regulations (Pilling, Brannon, Shanklin, Howells, & Roberts, 2008). Therefore, changing the behavior of employees in the organization and creating a food safety culture are very important actions to reduce food‐borne illness (Yiannas, 2009).

This study took the employees working in the institutional food service of schools as subjects with the purpose of studying whether their food safety behaviors in the workplace are affected by different leadership styles of managers. This study also explored the impact of leadership styles on organizational climate and further analyzed whether there is a link between organizational climate and employees' implementation of food safety and their hygiene behaviors in the hope of understanding the leadership style applicable to employees of institutional food services. This study can be a reference to the institutional food service sector for leading staff at the grassroots level.

## 2 LITERATURE REVIEW

### 2.1 Leadership style

Leadership is the ability of a leader to increase the effectiveness of the group (Northouse, 2013). In other words, a group of people with specific goals, when faced with challenges or conflicts, makes use of many different resources to satisfy the motivation of the members and reach goals (Burns, 1978). Maxwell (1998) stated that leadership is the process of leading members to achieve organizational goals. General leadership style is often divided into transactional leadership and transformational leadership. Transactional leaders trade or exchange valuable things (e.g., in the form of compensation, status, and other incentives) to encourage followers to achieve the most basic job performance; on the other hand, transformational leaders inspire and improve followers to achieve higher levels of performance than usual and to promote greater demand and desire among group members (Northouse, 2013). Transactional leaders and organization members agree on terms, and the leaders recognize and reward hard‐working employees and correct or punish employees for deviations or errors (Burns, 1978). Transformational leaders motivate employees to go beyond specific expectations (Doucet, Fredette, Simard, & Tremblay, 2015). Transformational leaders focus on promoting higher levels of motivation and morality for leaders and followers. This form of leadership is concerned with the needs and motives of followers and tries to help followers exert their maximum potential (Kouzes & Posner, 2012; Northouse, 2013). To adapt to a highly competitive ecological environment, an organization is bound to seek changes and innovations, and transformational leaders have a positive impact on successful organizational change (Ahmad & Gelaidan, 2011). Kreitner and Kinicki (2001) believe that transformational leadership achieves the process of interaction between leaders and subordinates by enhancing this relationship to the level of morality and motivation beyond the original contractual relationship, where the subordinates sincerely respect the leader and are willing to follow and obey, and the incentive actions of the leader cause the subordinates to accept the organization's vision and mission. Sheraz, Zaheer, Rehman, and Nadeem (2012) also state that transformational leaders can enhance the value of ethics in the workplace.

### 2.2 Organizational climate

Stringer (2002) advocated that organizational climate is a part of culture, and leadership behavior has the greatest impact on organizational climate. Organizational climate can be viewed as employees' perception of the working environment and therefore can be defined as the shared cognition of the organization and work environment by members of the organization (James et al., 2008). Organizational climate reflects the perceptions and emotions that members of the organization have about the nature of their work environment (Glisson & James, 2002). Leading the organization to establish a good organizational climate is a very important task for leaders (Ohly & Fritz, 2010). Organizational climate is also a key factor in determining employee behavior (Ball, Wilcock, & Colwell, 2010). As food preparation becomes increasingly commercial, the risk of potential food‐borne hazards in dining establishments due to poor food handling and hygiene is increasing, and changing employee behavior and creating a food safety culture in the organization are necessary considerations for reducing such hazards (Yiannas, 2009).

### 2.3 Food safety and hygiene

Food‐borne illness is a disease transmitted through the intake of infected foods and is one of the most widespread and significant public health problems (WHO, 2000). If a restaurant, a catered event, or a packed meal involves the slightest negligence in the manufacturing process, such as poor sanitation in dishes or packed meals, it can easily result in group food poisoning (Wall, de Louvois, Gilbert, & Rowe, 1996). Griffith, Livesey, and Clayton (2010) also estimates that approximately 70% of food poisoning cases occur in commercial food service places such as restaurants. Food‐borne illnesses associated with staff typically emerge one after another, and the frequency of occurrence appears to be increasing (Greig, Todd, Bartleson, & Michaels, 2007). The most common causes of illness include poor personal hygiene, cross‐contamination, and inadequate time/temperature (Guzewich & Ross, 1999). An employee's personal beliefs and attitudes toward consumer health and a sense of honor toward the job will affect the employee's food safety behavior (Pragle, Harding, & Mack, 2007).

### 2.4 School lunch institutional catering

School lunch institutional catering creates nonprofit group meals for schoolchildren (Mary & Gregoire, 2001). The catering mainly provides growing children with a nutritious health diet that is tasty, healthy, and safe, with balanced nutrition. It does not emphasize delicate ingredients; rather, eating can be finished within a fixed time, and the meal has a low cost with fixed timing, quantity, and pricing (Henroid & Sneed, 2004). Schools need adequate food safety training for food supply staff to ensure safe operation practices and to incorporate them into the school's food safety management plan (Curwood, Arendt, Rajagopal, & Stephen, 2017). Jipiu, Abdullah, Ariffin, Anuar, and Mohi (2016) showed that school dietary staff members have good knowledge and positive behavior to prevent cross‐contamination, perform hand cleaning procedures, maintain personal hygiene, and use disposable gloves. Although this study's results are satisfactory, it also clearly points out that food operators still lack food safety behaviors that would directly lead to the prevention of food‐borne diseases in schoolchildren.

According to the Centers for Disease Control and Prevention (2015), the three most common food safety misconducts that result in unsafe food and food‐borne illness are food handling errors, poor personal hygiene, and cross‐contamination. Painter et al. (2013) found that of the 17 food categories, the highest percentage (46%) cause of food‐borne diseases is the production process. This finding again demonstrates the importance of food safety training of school kitchen staff for food production and processing.

### 2.5 Relationships among leadership style, organizational climate, and food safety and hygiene behaviors

Cloete (2011) stated that organizational climate theory advocates that leaders in an organization have a significant influence on deciding organizational climate. If there are better leaders in the organization, then the organization will be more productive, more competitive, and more responsive (Griffith et al., 2010). Organizational climate is top‐down, starting with the superior and influencing the subordinate (Yianns, 2009). Lemons, Newsome, and Brashears (2013) pointed out that “leadership behavior directly affects organizational climate.” Eustace and Martins (2011) show that there is a strong positive correlation between heuristic leadership and organizational climate. On the other hand, transformational leadership can create an innovative organizational climate and increase employee creativity and satisfaction (Mohamed, 2016). Therefore, the author present H1 and H2: Transactional or transformational leadership styles have a direct impact on organizational climate.

Organizational climate is considered a collective attitude of employees toward the organization (Burton, Lauridsen, & Obel, 2004). Organizational climate is also formed through interactions between employees and affects the behavior of employees within the organization (Manning, Davidson, & Manning, 2005). Pragle et al. (2007) suggested that an organization's goals and expectations must be expressed clearly to employees, food safety training must be provided, strict regulations on food safety handling behaviors must be in place, and proper education must be provided to new employees so that the employees can become accustomed to food safety regulations upon entering the organization. Food safety is the behavior reflecting the food safety culture and food safety climate (Powell, Jacob, & Chapman, 2011). If an organization provides an environment of proper food safety behaviors and encourages employees to comply with food safety behaviors, then employees will perceive that the organization offers a positive food safety organizational climate. Employees will then have a positive will to comply with food safety behavior. Therefore, we have H3: Organizational climate has a positive impact on employees' compliance with food safety and hygiene behaviors.

Transformational leaders have a positive effect on employee self‐efficacy, motivation, creativity, and organizational performance (Bronkhorst, Steijn, & Vermeeren, 2015; Kim & Yoon, 2015; Newland, Newton, Podlog, Legg, & Tanner, 2015). Transactional leaders can increase job satisfaction and organizational identification (LePine, Zhang, Crawford, & Rich, 2015). Transformational leaders predict outcomes for individuals, groups, and organizations and make employees feel positive about job satisfaction through leadership behaviors of caring for employees (Wang, Oh, Courtright, & Colbert, 2011). Leaders should motivate employees, give positive feedback, and recognize good behaviors so that employees can be motivated to work in a safe and secure manner (Griffith et al., 2010). Therefore, we have H4 and H5: Transactional and transformational leadership styles have a direct impact on employees' compliance with food safety and hygiene behaviors.

**Figure 1:**
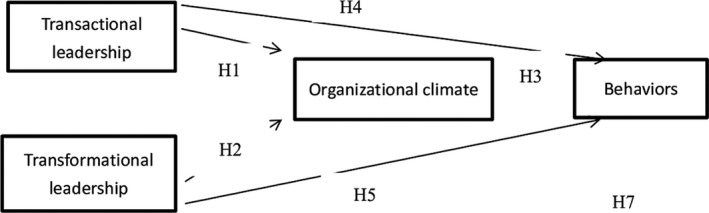
Research framework

Stringer (2002) proposed that “leadership behavior directly influences organizational climate.” Leaders provide employees with respect and trust by the leadership behavior of caring for employees, making employees' perceptual reaction to organizational climate better; on the other hand, transformational leaders can create an innovative organizational climate and increase employee creativity and employee satisfaction (Mohamed, 2016). Pragle et al. (2007) argued that the organization's goals and expectations must be expressed clearly to employees, food safety training must be provided, strict regulations on food safety handling behaviors must be in place, and proper education must be provided to new employees so that the employees can become accustomed to food safety regulations when entering the organization. Neal, Griffin, and Hart (2000) found that the organizational climate of the organization as a whole will significantly affect the safety climate in the workplace (safety climate is defined as a specific type of organizational climate, which describes the health and safety of the workplace as perceived by the employees). Safety climate is associated with employees' compliance with safety regulations and procedures in the workplace. If employees perceive that their organization treats them well and provides them with a positive organizational climate, then the employees will respond to the organization with positive attitudes and behaviors, such as more effort, more positive work‐related attitudes, and lower resignation rates (Aryee, Budhwar, & Chen, 2002). Hence, we have H6 and H7: Organizational climate is the mediating factor in transactional or transformational leadership styles for employees to comply with food safety and hygiene behaviors. The research framework is shown in Figure 1.

## 3 RESEARCH METHOD

### 3.1 Sample and data collection

This study took the employees working in institutional food service in northern Taiwan as the subjects. A quantitative survey was conducted using a questionnaire. With regard to the distribution of questionnaires, school dietitians in the northern part of Taiwan (Taoyuan City and New Taipei City) were asked to assist in forwarding the questionnaires. The questionnaires were distributed to employees who have worked in the school lunch institutional company for 3 months and who have already spent 8 hr in the food safety courses, including chefs and kitchen workers. The subjects are anonymous and voluntary to fill out the questionnaires. One hundred questionnaire copies were distributed for the trial and 100 copies were returned; 400 questionnaires were formally issued, and 324 copies were returned. The recovery rate is 81%.

### 3.2 Measures

The questionnaires for this study were designed with 5 constructs: transactional leadership, transformational leadership, organizational climate, food hygiene and safety behavior, and basic information.

#### 3.2.1 Leadership style scale

This study refers to the two leadership styles proposed by Burns (1978): transactional leadership and transformational leadership. The development of the questionnaire is for understanding the impact of these two leadership styles on the food safety and hygiene behaviors of the organizational climate and employees of institutional food services. The transactional leadership scale was adapted from Bass and Avolio (2004), with a total of five items; the transformational leadership scale was also modified from Lee, Almanza, Jang, Nelson, and Ghiselli (2013), with a total of four items.

#### 3.2.2 Organizational climate scale

The organizational climate scale was adapted from Lee et al. (2013). The seven items in this scale were revised and adjusted to meet the research purposes of this study and were designed to measure the organizational climate perceived by employees in the workplace.

#### 3.2.3 Scale of food safety and hygiene behaviors

The scale of food safety and hygiene behaviors was modified from Boeck, Jacxsens, Bollaerts, and Vlerick (2015). The items were adjusted to meet the research purposes of this study. The scale aims to measure the food safety and hygiene behaviors of the subjects with five items.

After the above questionnaires were revised and adjusted to meet the research purposes of this study, the wording was revised for clarity. The measurement was based on a 5‐point Likert scale, ranging from 5 (strongly agree) to 1 (strongly disagree), to measure the degree of consent of respondents to the items on the leadership style scale.

#### 3.2.4 Participants' demographic information

The participants' demographic information questionnaire includes gender, age, educational level, years of service, type of institutional food service, whether the employee has a license as a Chinese food technician, managers at the job site, daily servings for lunch, and number of kitchen employees.

### 3.3 Analysis methods

Five experts confirmed the content validity of this questionnaire. Cronbach's alpha values for reliability of transactional and transformational leadership styles were 0.880 and 0.818, respectively; Cronbach's alpha value for the organization climate was 0.893; and Cronbach's alpha value for the scale of food safety and hygiene behaviors was 0.918. This study used the SPSS statistical software package as a tool for data analysis. The data analysis methods include narrative statistics, Pearson correlation analysis, and regression analysis to examine the impact of leadership style on organizational climate, the impact of organizational climate on employees' compliance with food safety and hygiene behaviors, and the impact of leadership styles on employees' compliance with food safety and hygiene behaviors, and to explore the mediating impact of organizational climate.

## 4 RESULTS AND DISCUSSION

### 4.1 Participants' demographic information

More than 90% of the subjects in this study were women, and the age group was dominated by the older age group. Higher vocational school makes up the highest percentage of educational level. Most subjects had working years of over 1–4 years (inclusive), and more than one‐half of the subjects have the license of Chinese food technician; in addition, the highest proportion of main managers at the job site was kitchen foremen (41%), followed by dietitians (29.3%). School lunch facilities owned and operated by a public office accounted for 52.5%, while the other 47.5% were publicly owned under private operation. The highest proportion of average number of daily lunch servings was 1,501–2,000 (28.4%), followed by 1,001–1,500 (25.3%), and more than one‐half of the subjects had five–eight kitchen employees (51.5%; Table 1).

**Table 1:** Participant demographic information

Background variable	Item	Number	Percentage (%)
Gender	Male	32	9.9
	Female	292	90.1
Age	26–30 years old	8	2.5
	31–40 years old	61	18.8
	41–50 years old	86	26.5
	51–60 years old	144	44.4
	61 and over	25	7.7
Educational level	Junior high school or lower	110	34.0
	High school	66	20.4
	Vocational school	119	36.7
	Junior college	19	5.9
	College or higher	10	3.1
Seniority	<1 year	52	16.0
	1–4 years (inclusive)	96	29.6
	4–8 years (inclusive)	59	18.2
	8–12 years (inclusive)	39	12.0
	12–16 years (inclusive)	33	10.2
	16–20 years (inclusive)	33	10.2
	20 years or more	12	3.7
Is there a license for Chinese food technicians?	Yes	214	66.0
	No	110	34.0
Main manager on job site	Kitchen leader	133	41.0
	Chef	76	23.5
	Nutritionist	95	29.3
	Lunch secretary	11	3.4
	Other	9	2.8
School lunch method	Public office owned and operated (self‐administration)	170	52.5
	Public owned with private business operation (outsourcing)	154	47.5
Average daily servings for lunch	500 servings or less	11	3.4
	501–1,000 servings	19	5.9
	1,001–1,500 servings	82	25.3
	1,501–2,000 servings	92	28.4
	2,001–3,000 servings	36	11.1
	3,001 servings or more	25	7.7
Number of kitchen employees	1–4	55	17.0
	5–8	167	51.5
	9–12	66	20.4
	13 or more	36	11.1

### 4.2 Employee percentage of leaders' styles of organization climate and food safety behavior

According to the questions of transactional leadership style in Table 2, where the highest score is when the employee's performance is not good, the supervisor will clearly point out the problem; the second highest score is when the employee performs well, the supervisor will give positive feedback.

**Table 2:** Mean of transactional leadership style for employees

Construct	Items	Mean	*SD*	Ranking
Transactional leaders	1. When I am performing well, the supervisor will give me positive feedback	3.97	0.66	2
	2. When I have excellent performance, the supervisor will give a reward	3.94	0.91	4
	3. When I perform beyond my goal, the supervisor will give a compliment	3.96	0.90	3
	4. When my work is not up to standard, the supervisor will give a correction or punishment according to the circumstances	3.93	0.96	5
	5. When my job performance is poor, the supervisor will clearly point out the problem	4.14	0.81	1
Average		3.99	0.85	

Table 3 shows that the average mean of transformational leadership styles is between 3.81 and 4.16. The highest score is that the supervisors make employees feel that it is a pleasure to get along with them, working with them makes employees feel proud, and they encourage employees to reflect on past issues in new ways.

**Table 3:** Mean of transformational leadership style for employees

Construct	Items	Mean	*SD*	Ranking
Transformational leaders	1. My supervisors make me feel that it is a pleasure to get along with them, and working with them makes me feel proud	4.16	0.71	1
	2. My supervisors help me find the meaning of the work and explain in a few simple words what I could do and what I should do	3.98	0.71	3
	3. My supervisors encourage me to reflect on past issues in new ways	4.03	0.72	2
	4. My supervisors provide me with feedback on my job performance and help with my career planning	3.81	0.95	4
Average		3.99	0.77	

In terms of organizational climate, Table 4 shows that respondents have a positive view of the organizational climate in the units they serve (schools or institutional food service companies), including employees who are excited about their work and want to do their best for maximum performance, and every employee understands the goals of this company/school, while the supervisors strongly encourage employees to develop skills.

**Table 4:** Mean of organizational climate for employees

Construct	Items	Mean	*SD*	Ranking
Organizational climate	1. The supervisor will talk to the employee before making any changes affecting the employee and let the employee participate in the decision	4.05	0.74	4
	2. Employees receive adequate training. Supervisors strongly encourage employees to develop skills	4.07	0.66	3
	3. My company/school is very flexible and willing to use new ideas	4.02	0.68	6
	4. My company/school will use employee ideas to improve the employee's own work situation	3.92	0.75	7
	5. Every employee understands the goals of this company/school	4.10	0.61	2
Organizational climate	6. My company/school is very efficient and will not waste time and money	4.05	0.71	5
	7. Employees are excited about their work and want to do their best for maximum performance	4.12	0.67	1
Average		4.05	0.69	

The average number of behaviors complying with food safety according to Table 5 is between 4.30 and 4.44. We learn that most subjects in this study believe that they have observed food safety during their working hours and that they jointly observe food safety behaviors with others while setting a good example of behavior for others to follow and show their conscious behavior for compliance with food safety.

**Table 5:** Mean of food safety behavior for employees

Construct	Items	Mean	*SD*	Ranking
Food safety behaviors	1. During working hours, I have observed food safety	4.40	0.54	3
	2. During working hours, I will work with others to observe food safety behavior	4.44	0.53	1
	3. I will set a good example of following food safety behaviors for others to follow	4.41	0.59	2
	4. At the job site, I will actively remind or inform my colleagues about the rules and practices for compliance with food safety behaviors	4.39	0.56	4
	5. I will actively discuss things related to food safety behavior with my colleagues or supervisors	4.30	0.61	5
Average		4.39	0.57	

An analysis of transactional leadership style finds that most employees believe that when their job performance is not good, supervisors will clearly point out problems. At the same time, when they perform well, supervisors will give positive feedback. Thus, transactional leaders develop a tactical strategy and structure that contribute to the effectiveness of the organization, reward a subordinate's efforts, and correct a subordinate's mistakes and biased behaviors to achieve good organizational performance (Waldman, Ramirez, House, & Puranam, 2001). The World Health Organization (WHO, 2000) suggests that the education and training of food handlers and consumers are good means of preventing food‐borne diseases, because poor processing by food handlers causes the most food poisoning incidents. Therefore, safe food processing specifications should be followed and become familiar daily to form proper work habits. Transactional leaders will explain the work content and task requirements to employees and provide timely correction and guidance to let the employees understand the work methods and provide positive feedback to meet the needs of employees (Bass, 1985) to achieve food safety goals.

An analysis of transformational leadership style finds that most employees believe that it is a pleasure to get along with their supervisors and that working with supervisors makes employees feel proud—that is, transformational leaders encourage employees' motives to exceed specific expectations by influencing employees' beliefs and values (Doucet et al., 2015). Employees' compliance with food hygiene and safety behaviors is based not only on job requirements but also on the belief in maintaining the health of consumers (school staff and schoolchildren). Therefore, transformational leaders can inspire employees' sense of honor in their work and affect employees' actual compliance with food safety and hygiene behaviors. From an analysis of organizational climate, most employees feel that they are excited about their work and want to do their best for optimum performance. At the same time, every employee understands the goals of the company or institutional food service/school.

### 4.3 Influence relationship of each contract

This study uses Pearson correlation analysis to explore the correlation between the various constructs, as shown in Table 6. All facets were significantly positively correlated.

**Table 6:** Pearson correlation coefficients for all constructs

Constructs	Mean	*SD*	A1	A2	B	D
A1 Transactional leader	3.99	0.701	1			
A2 Transformational leader	4.00	0.628	0.682**	1		
B Organizational climate	4.05	0.540	0.587**	0.696**	1	
D Food safety and hygiene behaviors	4.39	0.491	0.251**	0.398**	0.477**	1

Note

*n* = 324.

** *p*<0.01

This study explored the effect of the transactional leadership style of managers of the institutional food service sector on organizational climate. The results show that the style of transactional leadership has a significant positive impact on organizational climate. The explanatory power reaches 34.2%; thus, H1 is supported, as shown in Table 7. This result indicates that the employees of institutional food services are aware that their supervisors exhibit a transactional leadership style and have a significant impact on organizational climate—that is, an influence on the shared cognition of their organization and the work environment of the employees of institutional food services. Organizational climate runs from top to bottom; it begins with superiors and influences subordinates (Yianns, 2009). The results of this study are also consistent with previous research whereby leadership behavior directly affects organizational climate (Lemons et al., 2013; Stringers, 2002).

**Table 7:** Regression analysis of transactional leadership style on organizational climate

	Unstandardized coefficient		Standardized coefficient	*T* value	*p* value
	Estimated value of *B*	*SE*	*β* distribution		
Transactional leadership style	0.452	0.035	0.587	13.006	0.000***

Note

*F* value: 169.152; *R* ^2^: 0.344; adjusted *R* ^2^: 0.342.

*** *p* < 0.001.

The results of transformational leadership also show a significant impact on organizational climate, and the explanatory power is as high as 48%; thus, H2 is supported, as shown in Table 8. The results of this study indicate that the employees of institutional food services are aware that their supervisor has a transformational leadership style that significantly affects the organizational climate. The results of this study are consistent with previous studies (Bruns, 1978; Hollander & Offermann, 1990; Lee et al., 2013; Zohar & Tenne‐Gazit, 2008). Some possible explanations are that employees frequently observe the words and deeds of their supervisors and have good interaction with their supervisors, and their supervisors share relevant organization policies and norms with employees and explain to employees the matters that should be observed and noted in the workplace, which gradually form the characteristics of the organization and are recognized by the employees. The results of this study lead to the conclusion that the establishment and maintenance of the organizational climate depend on the degree to which the employees understand the supervisory norms and the perceived relationship between them and their managers. Therefore, the relationship between transformational leadership and organizational climate as perceived by employees shows whether supervisors in the institutional food service industry can have transformational leadership. This form of leadership can thus facilitate frontline employees in the workplace in expressing the organizational climate they expect and want.

**Table 8:** Regression analysis of transformational leadership style on organization climate

	Unstandardized coefficient		Standardized coefficient	*T* value	*p* value
	Estimated value of *B*	*SE*	*β* distribution		
Transformational leadership style	0.599	0.034	0.696	17.410	0.000***

Note

*F* value: 303.111; *R* ^2^: 0.485; adjusted *R* ^2^: 0.483.

*** *p* < 0.001.

This study used organization climate as a self‐variant to explore the effect of organizational climate in the industry of institutional food service on an employee's compliance with food safety and hygiene behaviors. The results show that the impact of organizational climate on compliance with food safety and hygiene behaviors is significant, which means that organizational climate has a significantly positive impact on compliance with food safety and hygiene behaviors. The explanatory power is 22.5%; thus, H3 is supported, as shown in Table 9. The results of this study show that the perceived organizational climate of employees will significantly affect their food safety behavior in the workplace; in other words, if the employees perceive the organization's policies, norms, procedures, rewards, and support systems are clear, and the employees also feel that the organization is good to them, then the employees are more likely to put forth greater effort at work and observe food safety and hygiene behaviors in the workplace. Previous studies also support the relationship between organizational climate and employees' compliance with food safety and hygiene behaviors (Lee et al., 2013).

**Table 9:** Regression analysis of organizational climate on food safety and hygiene behaviors

	Unstandardized coefficient		Standardized coefficient	*T* value	*p* value
	Estimated value of *B*	*SE*	*β* distribution		
Organizational climate	0.434	0.045	0.477	9.741	0.000***

Note

*F* value: 94.878; *R* ^2^: 0.228; adjusted *R* ^2^: 0.225.

*** *p* < 0.001.

Measuring the impact of the transactional leadership style of managers in an institutional food service business on employees' compliance with food safety and hygiene behaviors, the results show that while transactional leadership style has a significant impact on compliance with food safety and hygiene behaviors, the explanatory power is only 6%. Although H4 is supported, it is less explanatory, as shown in Table 10.

**Table 10:** Regression analysis of transactional leadership style on food safety and hygiene behaviors

	Unstandardized coefficient		Standardized coefficient	*T* value	*p* value
	Estimated value of *B*	*SE*	*β* distribution		
Transactional leadership style	0.176	0.038	0.251	4.652	0.000***

Note

*F* value: 21.637; *R* ^2^: 0.063; adjusted *R* ^2^: 0.060.

*** *p* < 0.001.

The effect of a transformational leadership style in managers of an institutional food service business on employees' compliance with food safety and hygiene behaviors is shown to be significant (*β* = 0.398, *p* = 0.000) with an explanatory power of 15%, meaning that the transformational leadership style has a significantly positive impact on compliance with food safety and hygiene behaviors. Thus, H5 is supported, as shown in Table 11.

**Table 11:** Regression analysis of transformational leadership style on food safety and hygiene behaviors

	Unstandardized coefficient		Standardized coefficient	*T* value	*p* value
	Estimated value of *B*	*SE*	*β* distribution		
Transformational leadership style	0.311	0.040	0.398	7.794	0.000***

Note

*F* value: 60.747; *R* ^2^: 0.159; adjusted *R* ^2^: 0.156.

*** *p* < 0.001.

At the job site of an institutional food service business, supervisors and employees set up constructive transactions or exchanges to complete contractual tasks; supervisors describe job roles and task requirements and establish reward systems to fulfill employee expectations. Therefore, employees understand and work hard to achieve the goals of the organization while observing food safety and hygiene behaviors to ensure that the efforts they put forth are properly compensated. Previous research studies also support a significant influence of a transactional leadership style on employee performance (Masa'deh, Obeidat, & Tarhini, 2016).

Employees perceive the transformational leadership styles of supervisors as having a significant impact on their compliance with food safety in the workplace. Supervisors with a transformational leadership style exude an exemplary model of integrity and fairness, caring for and meeting the individual needs of employees and encouraging employees to develop new problem‐solving methods, etc. In this way, employees will be willing to exert extra effort to seek personal growth and reach the goals of the organization. Previous research also supports a significant relationship between a transformational leadership style and employee performance (Chi & Lai, 2011; Sheraz et al., 2012). There is also a positive and strong relationship between transformational leaders and safety compliance, safety participation, and safety attitudes of the employees (Mullen, Kelloway, & Teed, 2017).

Baron and Kenny (1986) stated the established mediation occurred under the following conditions: First, the independent variable must affect the mediator in the first equation; second, the independent variable must be shown to affect the dependent variable in the second equation; then, the mediator must affect the dependent variable in the third equation. If these conditions all hold in the predicted directions, then the effect of the independent variable on the dependent variable must be less in the third equation than in the second. Perfect mediation holds if the independent variable has no effect when the mediator is controlled. According to Table 12, a transactional leadership style significantly reduces such behavior, indicating that the influence of transactional leadership style on employees' compliance with food safety and hygiene behaviors generates complete mediating effects through organizational climate. Thus, H6 of this study is supported by empirical data, showing that transactional leadership styles have a direct impact on employee behavior.

**Table 12:** Regression analysis of transactional leadership style and organizational climate on food safety and health behavior

	Unstandardized coefficient		Standardized coefficient	*T* value	*p* value
	Estimated value of *B*	*SE*	*β* distribution		
Transactional leadership style	−0.031	0.042	−0.044	−0.732	0.465
Organizational climate	0.457	0.055	0.503	8.311	0.000***

Note

*F* value: 47.638; *R* ^2^: 0.229; adjusted *R* ^2^: 0.224.

*** *p* < 0.001.

Considering the impact of organizational climate on transformational leadership style under compliance with food safety and hygiene behaviors, Table 13 shows that the standardized coefficient beta value of transformational leadership style decreases from 0.398 to 0.128. The downward trend indicates that the transformational leadership style has a complete mediating effect on employees' compliance with food safety and hygiene behaviors through the organizational climate. Therefore, H7 is supported by empirical data.

**Table 13:** Regression analysis of transformational leadership style and organizational climate on food safety and hygiene behaviors

	Unstandardized coefficient		Standardized coefficient	*T* value	*p* value
	Estimated value of *B*	*SE*	*β* distribution		
Transformational leadership style	0.100	0.053	0.128	1.890	0.060
Organizational climate	0.352	0.062	0.388	5.702	0.000***

Note

*F* value: 49.605; *R* ^2^: 0.236; adjusted *R* ^2^: 0.231.

*** *p* < 0.001.

For the staff of the school lunch kitchen in the north, an independent checklist and related kitchen forms for daily lunch operations are in place. There are very detailed work practices, from the inspection of the ingredients to the postcleaning process. The leadership style perceived by the employees, whether it is transactional or transformational, significantly affects their compliance with food safety and hygiene behaviors. Therefore, this research hypothesis is supported by empirical data and can be established. Since there is an open communication channel between the employees of the institutional food service business and the main managers on the job site, the employees are happy to inform their supervisors of work recommendations and obtain positive feedback. The employees feel respected and supported. Therefore, they are more dedicated to their work and more willing to obey the leadership of the supervisor, with better compliance with food safety and hygiene behaviors. At the same time, supervisors and employees share and discuss organizational policies and norms with each other so that grassroots employees can speak out about the organizational climate they want, and they will find ways to maintain this climate. The results of this research are consistent with those of previous research—that is, leaders at the highest level create and develop the climate and tone of the organization, while leadership style is used to play the role of organizational climate and organizational culture advocate (Hollander & Offermann, 1990).

The results of this study indicate that organizational climate plays a very important role in an employee's compliance with food safety and hygiene behaviors. As noted earlier, organizational climate is a key factor in determining employee behavior (Ball et al., 2010). In addition to establishing food safety standards and procedures, supervisors can set up a positive organizational climate, including engaging employees in decisions, encouraging employees to receive training and skills, using new ideas, adopting employee ideas to improve their work, and making each employee understand the organization's goals—that is, establish values, beliefs, and work atmosphere that are shared with employees to create a food safety culture in the workplace, which in turn affects employees' behaviors in observing food safety.

## 5 CONCLUSION AND SUGGESTIONS

### 5.1 Conclusion

Leadership styles, organizational climate, and employees' compliance with food safety and hygiene behaviors are positively correlated. There is a significantly positive impact of transactional leadership style and transformational leadership style on organizational climate, there is a significantly positive impact of organizational climate on employees' compliance with food safety and hygiene behaviors, there is a significantly positive impact of transactional leadership style and transformational leadership style on employees' compliance with food safety and hygiene behaviors, and there is a significantly positive impact of transactional leadership style, transformational leadership style, and organizational climate on employees' food safety and hygiene behaviors. Organizational climate has a complete mediating effect between the transactional and transformational leadership style on food safety and hygiene behaviors. In other words, the employees working in institutional food service can enhance compliance with food safety and hygiene behaviors via the mediating effect of organizational climate.

### 5.2 Suggestions

#### 5.2.1 Suggestions on management of institutional food service

Ohly and Fritz (2010) found that leadership style has a significant impact on organizational climate, while organizational climate has a comprehensive impact on the organization because it impacts organizational performance. Therefore, it is recommended that supervisors of institutional food service strengthen their education and training of managers in school lunchtime kitchens and make the employees aware that leadership style and corporate/school system are good for building relationships between managers and employees on the job site.

The results of this study show that organizational climate has a significant impact on food safety and hygiene behaviors of employees of institutional food services. The study also finds that organizational climate is an intermediary factor in the leadership style of food safety and health behavior. Therefore, it is recommended that the supervisors of institutional food services should create a good organizational climate that is beneficial for employees to adhere to food safety and hygiene behaviors.

The results of this study show that leadership style has a significant impact on food safety and health behaviors of the employees of institutional food services. However, the difference between transactional leadership style and transformational leadership style is not significant. In other words, both leadership styles are applicable to employees of institutional food services; thus, management can use them freely to achieve organizational goals. With regard to the development of transactional and transformational leadership styles, it is recommended that the supervisors of institutional food services receive leadership training courses for empowerment training and have interschool/company visits for observations, exchanges, and experience sharing.

#### 5.2.2 Suggestions for lunch team of a company providing institutional food service/school—Creation of organizational climate

Leaders should engage employees when making decisions before making changes affecting employees. They should have good interaction with employees and make employees feel supported and respected. Employees should be given necessary education and training. In addition to the required health education according to regulations, employees can be encouraged to participate in relevant studies and develop skills, such as obtaining licenses as Chinese food technicians, taking small‐boiler training courses, cooking courses, and healthy eating courses, to enhance the value of their work, inject vigor into their work, and input competitiveness into their organization.

The company/school should remain flexible and be willing to employ new ideas. The company/school should review the relevant work regulations, conform to the trend of the times and regulations, encourage employees to receive education and training, and update or repair kitchen facilities and equipment timely. In addition, it should provide leadership‐style training courses for managers and make employees aware that the company/school has clear policies and flexible practices. Moreover, the school can actively seek funding from higher level units, including for equipment maintenance and renewal, employee salary subsidies, and grants for study courses. At the same time, additional integration activities can be added and transparency of company information can be increased to make employees work in a better environment, thus facilitating their compliance with food safety behaviors.

Employees can accurately understand the advantages and disadvantages of the workplace and related equipment operations and maintenance and make recommendations. The company/school should update or repair work equipment to meet the needs of employees at the right time and facilitate the smooth preparation of lunches. Since lunch preparation is very constrained, and work efficiency needs to be maintained to enable dining on time, a good and normal operation of the equipment is required under such time pressure, as much preparation time for employees is needed to comply with work norms, food safety, and hygiene behaviors.

The firm can also help employees be aware of business goals. When employees are immersed in hard work, they can be told about organizational goals. In addition to providing meals on time, the most important goal is to ensure safety and hygiene. To enable employees to understand the value of their work, the company/school needs to pass on experience through education and training.

#### 5.2.3 Suggestions for individuals of employees of institutional food services

It is recommended that employees should be rational in reflecting their suggestions for work or personal needs. They should respect the managers at the job site, follow normal communication channels to reflect their own opinions on appropriate occasions to create good interaction with supervisors, and actively participate in education and training and obtain relevant certificates.

#### 5.2.4 Suggestions for future research and limitations

A questionnaire survey method was adopted as the research method of this study. The depth and value of research results can be enhanced if long‐term observations or qualitative interviews are used for looking into the impact of leadership style and organizational climate on employee food safety and hygiene behaviors to further understand employees' views on leadership style and organizational climate.

Regulations require that 70% of staff working in institutional food service in school lunch kitchens must have a license as a Chinese food technician. Presently, it is not clear whether all institutional food service companies or schools are encouraging employees to obtain this license. However, some companies and schools encourage employees to actively obtain certification by way of subsidy fees and monthly incentives granted after obtaining the license. As previously indicated, employees who have food safety certifications have a significantly better attitude and intention regarding food safety and hygiene behaviors. At the same time, food safety certification presents a mediating effect (Lee et al., 2013). Therefore, it is suggested that in the future, whether or not an employee has a Chinese food technician license should be studied to investigate the related mediating effect of leadership style on food safety and health behaviors.

Some of the questionnaires in this study were distributed by the school dietitian to the staff of the institutional food service serving in the school. Because school dietitians are either direct managers or supervisors at the job site, the subjects might therefore be less willing to fill out their true thoughts or opinions. Some subjects might be affected by social desirability bias when filling out questionnaires and give answers they think correct are socially acceptable. An example is the first question in the scale of compliance with food safety behaviors: “I have observed food safety behaviors during my working hours.” This statement is considered socially acceptable—that is, everyone should comply with food safety regulations. Therefore, the employee may be inclined to agree or strongly agree with this statement, whether or not that person actually complies with food safety behaviors. This bias may thus affect the average mean of variables.

## CONFLICT OF INTEREST

The authors declare no conflict of interest.

## ETHICAL STATEMENTS

This study does not involve any human or animal testing. All of the subjects are anonymous and voluntary to fill out the questionnaires.

## References

[fsn31056-bib-0001] Ahmad, H. , & Gelaidan, H. M. (2011). Organizational culture, leadership styles, and employee’s affective commitment to change: A case of Yemen public sector. Journal of Organizational Management Studies, 2011, 2131–10.

[fsn31056-bib-0002] Andersen, J. A. (2016). An old man and the sea of leadership. Journal of Leadership Studies, 9(4), 70–81. 10.1002/jls.21422

[fsn31056-bib-0003] Aryee, S. , Budhwar, P. S. , & Chen, Z. X. (2002). Trust as a mediator of the relationship between organizational justice and work outcomes: Test of a social exchange model. Journal of Organizational Behavior, 23(3), 267–285. 10.1002/job.138

[fsn31056-bib-0004] Ball, B. , Wilcock, A. , & Colwell, S. (2010). *Factors predicting worker food safety behaviour in Ontario meat plants* . Poster session presented at the meeting of International Association for Food Protection, Anaheim, CA.

[fsn31056-bib-0005] Baron, R. M. , & Kenny, D. A. (1986). The moderator‐mediator variable distinction in social psychological research: Conceptual, strategic, and statistical considerations. Journal of Personality and Social Psychology, 51(6), 1173–1182. 10.1037/0022-3514.51.6.1173 3806354

[fsn31056-bib-0006] Bass, B. M. (1985). Leadership and performance beyond expectations. New York, NY: Macmillan.

[fsn31056-bib-0007] Bass, B. M. (1990). From transactional to transformational leadership: Learning to share the vision. Organizational Dynamics, 18(3), 19–31. 10.1016/0090-2616(90)90061-S

[fsn31056-bib-0008] Bass, B. M. , & Avolio, B. J. (2004). Multifactor leadership questionnaire, 3rd ed Menlo Park, CA: Mind Garden.

[fsn31056-bib-0009] Bennis, W. G. , & Nanus, B. (1985). Leaders: The strategies for taking charge. New York, NY: Harper and Row.

[fsn31056-bib-0010] Boeck, E. D. , Jacxsens, L. , Bollaerts, M. , & Vlerick, P. (2015). Food safety climate in food processing organizations: Development and validation of a self‐assessment tool. Trends in Food Science and Technology, 46, 242–251. 10.1016/j.tifs.2015.09.006

[fsn31056-bib-0011] Burton, R. M. , Lauridsen, J. , & Obel, B. (2004). The impact of organizational climate and strategic fit on firm performance. Human Resource Management, 43(1), 67–82.

[fsn31056-bib-0012] Bronkhorst, B. , Steijn, B. , & Vermeeren, B. (2015). Transformational leadership, goal setting, and work motivation: The case of a Dutch municipality. Review of Public Personnel Administration, 35(2), 124–145. 10.1177/0734371X13515486

[fsn31056-bib-0013] Burns, J. M. (1978). Leadership. New York, NY: Harper & Row.

[fsn31056-bib-0014] Center for Disease Control and Prevention (2015). Preventing future outbreaks. Retrieved from http://www.cdc.gov/foodsafety/outbreaks/preventioneducation/future.html

[fsn31056-bib-0015] Chi, L. C. , & Lai, C. C. (2011). A research on the influence of leadership style and job characteristics on job performance among accountants of county and city government in Taiwan. Public Personnel Management, 40(2), 101–118. 10.1177/009102601104000202

[fsn31056-bib-0016] Cloete, M. (2011). *The relationship between leadership styles and organisational climate* . Master's dissertation.University of South Africa, Pretoria.

[fsn31056-bib-0017] Curwood, S. C. , Arendt, S. W. , Rajagopal, L. , & Stephen, S. W. (2017). Challenges to implementing food safety and produce handling training in school meal program. Journal of Food Studies, 6(1), 31 10.5296/jfs.v6i1.11669

[fsn31056-bib-0018] Doucet, O. , Fredette, M. , Simard, G. , & Tremblay, M. (2015). Leader profiles and their effectiveness on employee outcomes. Human Performance, 28(3), 244–264.

[fsn31056-bib-0019] Eustace, A. , & Martins, N. (2011). The role of leadership in shaping organizational climate: An example from the fast moving consumer goods industry. Journal of Industrial Psychology, 40(1). 10.4102/sajip.v40.1112

[fsn31056-bib-0020] Glisson, C. , & James, L. R. (2002). The cross level effects of culture and climate in human service teams. Journal of Organizational Behavior, 23(6), 767–794.

[fsn31056-bib-0021] Greig, J. D. , Todd, E. C. D. , Bartleson, C. A. , & Michaels, B. S. (2007). Outbreaks where food workers have been implicated in the spread of foodborne disease. Part 1. Description of the problem, methods, and agents involved. Journal of Food Protection, 70(7), 1572–1761. 10.4315/0362-028x-70.7.1752 17685355

[fsn31056-bib-0022] Griffith, C. J. , Livesey, K. M. , & Clayton, D. (2010). The assessment of food safety culture. British Food Journal, 112, 439–456.

[fsn31056-bib-0023] Guzewich, J. , & Ross, M. P. (1999). *Evaluation of risks to microbiological contamination of ready‐to eat food by food preparation workers and the effectiveness of intervention to minimize those risks* . Food and Drug Administration Center for Food Safety and Applied Nutrition Retrieved from http//vm.cfsan.fda.gov/~ear/-rterisk.html

[fsn31056-bib-0024] Henroid, D. Jr , & Sneed, J. (2004). Readiness to implement hazard analysis and critical control point (HACCP) systems in Iowa schools. Journal America Dietetic Association, 104(2), 180–185. 10.1016/j.jada.2003.11.009 14760564

[fsn31056-bib-0025] Hollander, E. P. , & Offermann, L. R. (1990). Power and leadership in organizations; relationship in transition. American Psychologist, 45(2), 179–189.

[fsn31056-bib-0026] James, L. R. , Choi, C. C. , Ko, C. H. E. , McNeil, P. K. , Minton, M. K. , Wright, M. A. , & Kim, K. I. (2008). Organizational and psychological climate: A review of theory and research. European Journal of Work and Organizational Psychology, 17(1), 5–32.

[fsn31056-bib-0027] Jipiu, L. B. , Abdullah, R. P. S. R. , Ariffin, H. F. , Anuar, N. A. M. , & Mohi, Z. (2016). Good hygiene practices (GHP) among school canteen food handlers In Radzi S. M., Mohd Hanafiah M. H., Sumarjan N., Mohi Z., Sukyadi D., Suryadi K. & Purnawarman P. (Eds.), Heritage, culture and society: Research agenda and best practices in the hospitality and tourism industry ‐ Proceedings of the 3rd International Hospitality and Tourism Conference, IHTC 2016 and 2nd International Seminar on Tourism, ISOT 2016 (pp. 723–728). London, UK: CRC Press/Balkema.

[fsn31056-bib-0028] Kim, S. , & Yoon, G. (2015). An innovation‐driven culture in local government: Do senior manager׳s transformational leadership and the climate for creativity matter? Public Personnel Management, 44(2), 147–168. 10.1177/0091026014568896

[fsn31056-bib-0029] Kouzes, J. M. , & Posner, B. Z. (2012). The leadership challenge, 5th ed San Francisco, CA: Wiley.

[fsn31056-bib-0030] Kreitner, R. , & Kinicki, A. (2001). Organizational behavior. Boston, MA: Irwin/McGraw‐Hill.

[fsn31056-bib-0031] Lee, J. E. , Almanza, B. A. , Jang, S. , Nelson, D. C. , & Ghiselli, R. F. (2013). Does transformational leadership style influence employees’ attitudes toward food safety practice? International Journal of Hospitality Management, 33, 282–293.

[fsn31056-bib-0032] Lemons, L. , Newsome, S. , & Brashears, T. (2013). *An investigation of the effect of leadership change on organizational climate* . Poster proceedings of the (2013). American Association for Agricultural Education Research Conference (pp. 195–198). OH: Columbus.

[fsn31056-bib-0033] LePine, M. A. , Zhang, Y. , Crawford, E. R. , & Rich, B. L. (2015). Turning their pain to gain: Charismatic leader influence on follower stress appraisal and job performance. Academy of Management Journal, 59(3), 1036–1059. 10.5465/amj.2013.0778

[fsn31056-bib-0034] Manning, M. L. , Davidson, M. , & Manning, R. L. (2005). Measuring tourism and hospitality employee workplace perceptions. International Journal of Hospitality Management, 25, 75–90. 10.1016/j.ijhm.2004.05.001

[fsn31056-bib-0035] Mary, F. N. , & Gregoire, M. B. (2001). Analysis of the process used to select a food production system for school foodservice. Journal Child Nutrition and Management, 24(2), 84–91.

[fsn31056-bib-0036] Masa'deh, R. , Obeidat, B. Y. , & Tarhini, A. (2016). A Jordanian empirical study of the associations among transformational leadership, transactional leadership, knowledge sharing, job performance, and firm performance: A structural equation modelling approach. Journal of Management Development, 35(5), 681–705. 10.1108/JMD-09-2015-0134

[fsn31056-bib-0037] Maxwell, J. C. (1998). The 21 irrefutable laws of leadership : Follow them and people will follow you. Nashille, Tenessee: Thomas Nelson Inc.

[fsn31056-bib-0038] Mohamed, L. M. (2016). Assessing the effects of transformational leadership: A study on Egyptian hotel employees. Journal of Hospitality and Tourism Management, 26, 49–59. 10.1016/j.jhtm.2016.04.001

[fsn31056-bib-0039] Mohan, T. (2000). Leadership Styles in Information Technology Projects. International Journal of Project Management, 18(4), 235–241.

[fsn31056-bib-0040] Mullen, J. , Kelloway, K. , & Teed, M. (2017). Employer safety obligations, transformational leadership and their interactive effects on employee safety performance. Safety Science, 91, 405–412. 10.1016/j.ssci.2016.09.007

[fsn31056-bib-0041] Neal, A. , Griffin, M. A. , & Hart, P. M. (2000). The impact of organizational climate on safety climate and individual behavior. Safety Science, 34(1–3), 99–109. 10.1016/S0925-7535(00)00008-4

[fsn31056-bib-0042] Newland, A. , Newton, M. , Podlog, L. , Legg, W. E. , & Tanner, P. (2015). Exploring the nature of transformational leadership in sports: A phenomenological examination with female athletes. Qualitative Research in Sport, Exercise and Health, 7(5), 663–687.

[fsn31056-bib-0043] Northouse, P. G. (2013). Leadership: Theory and practice. Thousand Oaks, CA: Sage.

[fsn31056-bib-0044] Ohly, S. , & Fritz, C. (2010). Work characteristics, challenge appraisal, creativity, and proactive behavior: A multilevel study. Journal of Organizational Behavior, 31, 543–565. 10.1002/job.633

[fsn31056-bib-0045] Painter, J. A. , Hoekstra, R. M. , Ayers, T. , Tauxe, R. V. , Braden, C. R. , Angulo, F. J. , & Griffin, P. M. (2013). Attribution of foodborne illnesses, hospitalizations, and deaths to food commodities by using outbreak data, United States, 1998–2008. Emerging Infectious Disease Journal, 19(3), 407–415. 10.3201/eid1903.111866 PMC3647642 23622497

[fsn31056-bib-0046] Pilling, V. K. , Brannon, L. A. , Shanklin, C. W. , Howells, A. D. , & Roberts, K. R. (2008). Identifying specific beliefs to target to improve restaurant employees’ intentions for performing three important food safety behaviors. Journal of the American Dietetic Association, 108(6), 991–997. 10.1016/j.jada.2008.03.014 18502232

[fsn31056-bib-0047] Powell, D. A. , Jacob, C. J. , & Chapman, B. J. (2011). Enhancing food safety culture to reduce rates of foodborne illness. Food Control, 22, 817–822. 10.1016/j.foodcont.2010.12.009

[fsn31056-bib-0048] Pragle, A. S. , Harding, A. K. , & Mack, J. C. (2007). Food workers’ perspectives on handwashing behaviors and barriers in the restaurant environment. Journal of Environmental Health, 69(10), 27–32. 17583293

[fsn31056-bib-0049] Sheraz, A. , Zaheer, A. , Rehman, K. , & Nadeem, M. (2012). Enhancing employee performance through ethical leadership, transformational leadership and organizational culture in development sector of Pakistan. African Journal of Business Management, 6, 1244–1251.

[fsn31056-bib-0050] Stringer, R. (2002). Leadership and organizational climate. Upper Saddle River, NJ: Prentice Hall.

[fsn31056-bib-0051] Waldman, D. , Ramirez, G. , House, R. , & Puranam, P. (2001). Does leadership matter? CEO leadership attributes and profitability under conditions of perceived environmental uncertain. Academy of Management Journal, 44, 134–143.

[fsn31056-bib-0052] Wall, P. G. , de Louvois, J. , Gilbert, R. J. , & Rowe, B. (1996). Food poisoning: Notifications, laboratory reports and outbreaks—where do the statistics come from and what do they mean. Communicable Disease Report, 6, 93–100. 8680502

[fsn31056-bib-0053] Wang, G. , Oh, I. S. , Courtright, S. H. , & Colbert, A. E. (2011). Transformational leadership and performance across criteria and levels: A meta‐analytic review of 25 years of research. Group and Organization Management, 36(2), 223–270. 10.1177/1059601111401017

[fsn31056-bib-0054] World Health Organization (2000). Foodborne disease: A focus for health education. Geneva, Switzerland: World Health Organization.

[fsn31056-bib-0055] Yiannas, F. (2009). Food safety culture: Creating a behavior‐based food safety management system. Spring Street, NY: Springer Science.

[fsn31056-bib-0056] Zohar, D. , & Tenne‐Gazit, O. (2008). Transformational leadership and group interaction as climate antecedents: A social network analysis. Journal of Applied Psychology, 93(4), 744–757. 10.1037/0021-9010.93.4.744 18642981

